# Topical Use of Sucralfate in Cutaneous Wound Management: A Narrative Review with a Veterinary Perspective

**DOI:** 10.3390/vetsci12080756

**Published:** 2025-08-13

**Authors:** Lucrezia Accorroni, Fabrizio Dini, Nicola Pilati, Andrea Marchegiani, Marilena Bazzano, Andrea Spaterna, Fulvio Laus

**Affiliations:** School of Biosciences and Veterinary Medicine, University of Camerino, Via Circonvallazione 93/95, 62024 Matelica, MC, Italy; fabrizio.dini@unicam.it (F.D.); nicola.pilati@unicam.it (N.P.); andrea.marchegiani@unicam.it (A.M.); marilena.bazzano@unicam.it (M.B.); andrea.spaterna@unicam.it (A.S.); fulvio.laus@unicam.it (F.L.)

**Keywords:** skin, sucralfate, topical, wound

## Abstract

Wound management is a fundamental skill for veterinarians, requiring a systematic approach to wound care and a deep understanding of the biological principles underlying healing. Sucralfate, widely known as a mucoprotective agent for gastroduodenal ulcers, has recently shown promising topical effects in human skin lesions by binding and protecting growth factors from proteolytic degradation, thereby enhancing their local availability. This action promotes angiogenesis, chemotaxis and cell proliferation, while reducing oxidative stress and exerting bacteriostatic and bactericidal effects against common pathogens. However, the veterinary-specific literature on topical sucralfate is extremely limited, with most available data derived from experimental studies in rodent and porcine models, rather than clinical studies in common veterinary species. Nonetheless, these preliminary studies suggest a potential role for sucralfate in accelerating the healing process through improved collagen synthesis, neovascularization and fibroblast activity. Given the species-specific challenges in veterinary wound healing—especially in horses and cats, prone to delayed healing and exuberant granulation tissue—sucralfate represents a promising, cost-effective and safe candidate for clinical use. This narrative review synthetizes current evidence on sucralfate’s mechanisms and therapeutic benefits across human and veterinary contexts, highlighting the need for controlled, multidisciplinary veterinary studies. Validating sucralfate’s efficacy in clinical settings could enable the growing owner demand for advanced care to be satisfied, shorten recovery times, reduce complications and improve animal welfare.

## Simple Summary

Wound healing in veterinary medicine can be slow and complicated. Sucralfate, commonly known for the treatment of gastroduodenal and esophageal ulcers, has recently shown promising effects as a topical agent in improving skin wound healing. It supports tissue repair by preserving growth factors, reducing inflammation, controlling infections and relieving pain by forming a protective barrier over exposed nerve endings. Although most studies have been conducted in humans and experimental animals, sucralfate may offer valuable benefits in veterinary patients, especially horses and cats. This review summarizes current evidence and highlights the need for further clinical research.

## 1. Introduction

The skin serves as a physical, chemical and bacterial defensive barrier, protecting vertebrates’ bodies from the external environment, making wound healing essential for survival [1,2,3]. Any skin injury disrupts its integrity, impairing function and potentially leading to disability or death [2]. Immediately after injury, a complex cascade begins to restore homeostasis and repair tissue, involving three overlapping phases: inflammatory, proliferative and remodeling [2,4,5]. The inflammatory phase starts with vascular responses, causing hemostasis, followed by leukocyte infiltration, including neutrophils, monocytes and lymphocytes [5]. Monocytes mature into macrophages, whose polarization into pro-inflammatory (M1) or pro-regenerative (M2) types is crucial in progressing to the proliferative phase [6]; in particular, pro-regenerative macrophages secrete growth factors (GFs) that stimulate cell proliferation and matrix synthesis [5].

During the proliferative phase, fibroblasts, endothelial cells and keratinocytes multiply, driving granulation tissue formation, fibroblast accumulation, neovascularization and reepithelialization [5]. Key growth factors, alongside collagen, contribute to tissue repair [6]. The maturation phase remodels the extracellular matrix through protein synthesis and degradation, resulting in organized scar tissue formation [7].

In veterinary medicine, skin disorders—including wounds—account for nearly a quarter of the entire case load in clinical practice [8]. These data highlight the need for improved strategies in the management of dermatological conditions. The skin represents the largest organ in the animal body, and disorders can be triggered not only by inadequate nutrition and/or hormonal imbalances but also by a wide range of other factors, including microorganisms, flea infestations, bacterial infections, physical or chemical agents and immunological reactions, all capable of initiating and perpetuating dermatological disorders [8,9,10,11].

Wound management is a fundamental skill for veterinarians, and successful treatment requires accurate knowledge of the biochemistry of the healing process and the various factors that can delay or prevent it, as well as a thorough understanding of the wide range of products available to treat this pathological condition and the mechanisms of action [12].

There are several conventional medication options for cutaneous wounds in animals, and, recently, a few reviews—mainly focused on human medicine—have examined the effectiveness of topical sucralfate, compared to other molecules, as an alternative treatment for various skin pathological conditions [13,14].

The topical use of sucralfate in veterinary medicine has so far been largely limited to experimental studies, often involving animal models with induced systemic diseases (e.g., diabetes mellitus), primarily to support its potential applications in human medicine, where such conditions are known to delay or impair normal wound healing [5]. Nonetheless, wound healing remains a critical and costly concern in animal practice, and inadequate management can result in delayed recovery, complications or permanent tissue damage [5]. Despite the limited clinical use, the pharmacological properties of sucralfate—particularly its anti-inflammatory, cytoprotective and tissue-regenerative effects—suggest a promising role in veterinary wound care.

The objectives of this paper are to review the recent studies that provide evidence- based information concerning the use of sucralfate in the treatment of cutaneous wounds in animals and to highlight its mechanisms of action and safety.

For this narrative review, the following research platforms were used: PubMed and Google Scholar. Literature searches were carried out using the following keywords: “veterinary” and/or “animal”; “wound” and/or “skin”; “cutaneous” and/or “topical”; and “sucralfate”. These were used in the databases following their syntax rules. Publications were selected without date restrictions. The inclusion criteria comprised randomized controlled trials, qualitative studies and reviews. All animal species, including experimental models, were included to provide a general comprehensive overview of evidence on the topic. The exclusion criteria were articles published in a language other than English and/or those not peer-reviewed. A total of 470 articles were retrieved. After excluding those that did not address sucralfate as a treatment methodology for skin wounds, 58 articles remained. Subsequently, following the application of the exclusion criteria, a final total of 36 publications were included in the review.

## 2. Sucralfate’s Mechanism of Action

**Figure 1:**
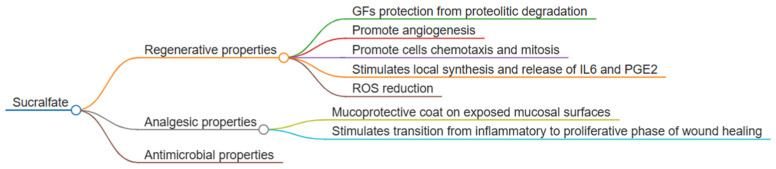
Schematic representation of sucralfate’s mechanism of action.

**Figure 2:**
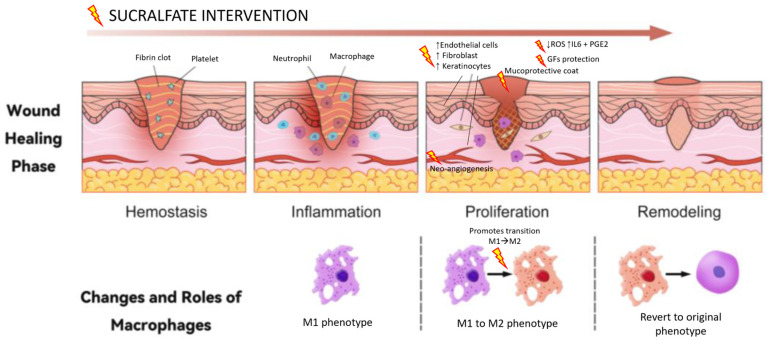
Mechanisms of wound healing and sucralfate intervention. Figure adapted from Li, J. et al., 2024 [30]. Licensed under CC BY 4.0.

Sucralfate is a basic complex salt of a disaccharide, namely sucrose octasulfate, and aluminum hydroxide; it is commonly used as a mucoprotective agent to prevent and treat gastroduodenal ulcers and gastritis in both humans [15] and animals [16,17]. Under low pH conditions, it forms a polyanionic gel-like barrier that binds to proteins in the ulcer crater, thereby protecting the tissue, increasing mucus production and hydrophobicity and binding bile acids [6,17,18]. A schematic overview of sucralfate’s mechanisms of action and its modulatory effects on wound healing is provided in Figure 1 and Figure 2, respectively.

Each phase of wound healing involves a complex cascade of microscopic events regulated by mediators such as growth factors, cytokines and chemokines [5]. Sucralfate’s active component, sucrose octasulfate, interacts strongly with several growth factors, binding and protecting them from proteolytic degradation and thus enhancing their local availability [6,18]. Its most significant contribution occurs during the proliferative phase [19], where it binds to fibroblast growth factor (FGF) and epidermal growth factor (EGF), promoting angiogenesis and enhancing chemotaxis and mitosis in fibroblasts, endothelial cells, mesenchymal cells and keratinocytes [19].

In addition, sucralfate stimulates the local synthesis and release of interleukin-6 (IL-6) by fibroblasts and prostaglandin E2 (PGE2) in basal keratinocytes, which further supports increased blood flow, mitotic activity and epithelial cell migration [20,21,22]. It also plays a protective role by reducing the levels of reactive oxygen species (ROS), which, when not neutralized by antioxidants, can perpetuate tissue damage and impair wound healing [17,19,23].

Sucralfate’s ability to coat and protect exposed mucosal surfaces, including those with open nerve endings or raw muscle, helps to accelerate the transition from the inflammatory to the proliferative phase. This property has been shown to reduce postoperative pain following procedures such as hemorrhoidectomy and tonsillectomy, thereby lowering the need for analgesics [17,24,25,26,27].

In addition to its regenerative and analgesic effects, sucralfate exhibits antimicrobial properties. Early human studies demonstrated its bactericidal effects against 68 out of 128 Gram-negative bacilli strains and bacteriostatic activity against all tested strains. Moreover, inhibitory effects were observed against *Pseudomonas aeruginosa* at pH 6.0 and 7.4 and against *Escherichia coli* and *Enterococcus faecalis* at pH 6.0. Sucralfate also enhanced the antibacterial efficacy of antibiotics against *Helicobacter pylori*, suggesting a synergistic effect [17,28]. Because infection prevention and management are critical to optimal wound healing, these findings are particularly relevant. A recent in vitro pilot study demonstrated the efficacy of a sucralfate formulation against common veterinary cutaneous pathogens, including *Escherichia coli*, *Enterococcus faecalis* and *Staphylococcus pseudointermedius*—a leading cause of superficial skin infections in dogs and cats [29].

## 3. Sucralfate’s Topical Use in Human Medicine

Topical sucralfate has been extensively studied for its wound healing and anti-inflammatory properties across various human clinical conditions. Its efficacy is well documented in second-degree burn wounds, where multiple randomized controlled trials and systematic reviews indicate that sucralfate not only accelerates epithelialization and reduces infection rates but also improves patient comfort and reduces the healing time when compared to traditional agents such as silver sulfazidine [31,32,33]. For instance, Godhi et al. (2017) [32] reported significantly faster healing and better pain management in patients treated with topical sucralfate compared to silver sulfazidine, emphasizing sucralfate’s role in promoting collagen synthesis and neovascularization.

In mucocutaneous inflammatory conditions, including post-radiotherapy mucositis and dermatitis, sucralfate’s mucoprotective and anti-inflammatory effects have been shown to significantly reduce symptom severity and duration [34,35,36]. These studies suggest that sucralfate forms a physical barrier that shields damaged mucosae from irritants and supports tissue regeneration, reducing the incidence of treatment interruptions in oncology patients.

The management of anorectal diseases has also benefited from topical sucralfate application. Several clinical trials demonstrate that sucralfate creams and suppositories reduce postoperative pain and promote faster wound healing after hemorrhoidectomy and in chronic hemorrhoidal disease, likely through enhanced epithelial repair and localized anti-inflammatory effects [13,20,37]. Meta-analyses confirm a consistent reduction in pain scores and improved wound healing metrics, making sucralfate a valuable adjunct therapy in proctologic surgery.

In pediatric dermatology, topical sucralfate shows beneficial effects in treating irritant diaper dermatitis. Systematic reviews confirm that sucralfate ointments reduce inflammation and promote faster barrier recovery compared to placebos, without significant adverse effects [14,38,39].

The role of topical sucralfate in chronic ulcer management—including venous ulcers, diabetic foot ulcers, pressure ulcers and other non-healing wounds—has been explored in various randomized and observational studies. Results consistently show that sucralfate enhances granulation tissue formation, stimulates local fibroblast proliferation and promotes angiogenesis through the stabilization of growth factors, resulting in accelerated wound contraction and epithelialization [40,41,42,43,44]. These benefits translate into improved clinical outcomes, including reduced wound sizes and faster closure times, which are critical in reducing the infection risk and morbidity.

Additionally, sucralfate’s analgesic properties have been validated in multiple surgical and wound care settings. Clinical trials report significant reductions in pain severity following hemorrhoidectomy, tonsillectomy and oral surgeries when treated with topical sucralfate compared to placebos or saline rinses [45,46,47,48]. These effects are attributed to sucralfate’s capacity to coat exposed nerve endings and reduce local inflammation, thereby improving patient comfort and recovery.

A summary of the principal clinical studies evaluating topical sucralfate for various mucocutaneous conditions in human medicine is provided in Table 1. Overall, sucralfate’s unique mechanism—combining growth factor protection, mucosal coating and anti-inflammatory action—supports its broad applicability in wound and inflammatory mucocutaneous disease management. However, further studies are necessary to optimize the therapeutic protocols, confirm its long-term efficacy and assess its comparative effectiveness against other emerging wound care agents.

Across the reviewed articles, wound healing was evaluated using clinical parameters such as the lesion size and healing time, granulation tissue, epithelialization, pain and exudate, often assessed with semiquantitative scales or standardized tools like the Pressure Ulcer Scale for Healing (PUSH) score. In some cases, histopathological analysis was also performed on biopsy samples stained with hematoxylin–eosin or other specific methods, evaluating fibroblast activity, inflammation, neovascularization and reepithelialization. Statistical analyses were commonly conducted using the SPSS software (version 15) and/or applying appropriate tests, including ANOVA, Student’s *t*-test, Kruskal–Wallis and chi-squared. A significance level of *p* < 0.05 was typically adopted [22,24,33,40]. Despite differences in methodology, these assessment tools enabled a systematic and comparative evaluation of sucralfate’s therapeutic effects.

## 4. Topical Sucralfate’s Experimental Use in Veterinary Medicine

Few studies have investigated the topical application of sucralfate in veterinary medicine, and all currently available data are limited to experimental animal models—primarily pigs and small rodents. These animals are commonly used as translational models for human wound healing, providing valuable comparative insights [5]. A summary of the main experimental studies evaluating the topical application of sucralfate in animal wound models is presented in Table 2.

Yildizhan et al. [18] evaluated the effects of the daily topical application of 10% sucralfate cream on 2 cm full-thickness dorsal skin wounds in rats. Their findings showed that sucralfate significantly accelerated wound healing by enhancing neovascularization, collagen density and organization, fibroblast activation and reepithelialization. Importantly, epidermal growth factor (EGF) expression was markedly improved in the sucralfate-treated group. Statistically significant differences in wound closure were observed on days 7, 14 and 21 compared to the control group treated with saline (*p* ≤ 0.05), confirming the superior recovery outcomes in the sucralfate group.

Beheshti et al. [49] also demonstrated the wound healing potential of topical sucralfate by comparing it to silver sulfadiazine in a rat model of second-degree burns. Their study showed that sucralfate led to complete wound healing in 100% of cases, outperforming silver sulfadiazine (91%) and cold cream (76%) and promoting full epidermal regeneration and tissue repair.

Le et al. [6] introduced an innovative approach using bioactive sucralfate-based microneedles to deliver interleukin-4 (IL-4) in both porcine and diabetes-induced murine models. Their results demonstrated that IL-4 facilitated macrophage polarization toward a pro-regenerative phenotype, while sucralfate stabilized growth factors by preventing proteolytic degradation. This dual action promoted cell proliferation, angiogenesis and enhanced wound healing. Animals treated with IL-4 + sucralfate microneedles exhibited the fastest macroscopic wound closure, a higher collagen intensity, accelerated cell growth and more rapid angiogenesis compared to all control groups.

In another study, Yuniati at al. [19] assessed the efficacy of sucralfate, platelet-rich plasma (PRP) and their combination in treating diabetic ulcers in rats. Although the combination therapy proved most effective, sucralfate alone significantly stimulated fibroblast activity, increased the local concentrations of growth factors, promoted angiogenesis and enhanced macrophage-mediated tissue remodeling. These effects collectively contributed to improved wound contraction and reepithelialization.

More recently, Yaşar et al. [22] investigated the application of 10% sucralfate cream in treating radiofrequency-induced burns in rats. Early observations indicated increased vasodilation and leukocyte chemotaxis in the sucralfate group, suggesting a more intense initial inflammatory response. However, from day 14 onwards, sucralfate-treated wounds exhibited markedly higher levels of angiogenesis, reepithelialization, total collagen synthesis and fibroblast proliferation. By the fourth week, the granulation tissue was significantly thicker in the sucralfate group, underlining its role in enhancing the proliferative and remodeling phases of healing.

## 5. Discussion: Challenges and the Potential Role of Topical Sucralfate

Wound healing in animals is a complex physiological process, often accompanied by various complications [12,50]. In horses, cutaneous injuries are particularly common and represent a significant economic burden for owners. This is largely due to the frequent requirement for healing by secondary intention, which substantially prolongs recovery times [7,50]. Granulation tissue formation by fibroblasts is a key component of successful cutaneous wound healing. However, fibroproliferative disorders—characterized by the excessive deposition of granulation tissue and extracellular matrix—commonly complicate wound management in horses, particularly when the lesions are located on the distal limbs [50,51]. Notably, while wounds on the trunk and distal neck typically heal through contraction, wounds on the proximal neck, head and limbs primarily close by reepithelialization, similar to the process observed in humans [7,51]. Horses exhibit a unique inflammatory response marked by the deficient leukocyte-mediated production of cytokines and chemoattractants, resulting in a prolonged and ineffective initial phase of healing [5]. Anatomical factors of the distal limb—including limited soft tissue coverage, reduced vascular supply, high mobility and exposure to environmental contaminants—further contribute to the pathogenesis of exuberant granulation tissue [5,50,52]. Additionally, dysregulated growth factor levels and disorganized collagen deposition exacerbate the problem.

Sucralfate has demonstrated a beneficial role in full-thickness skin wound healing, where it promotes epithelial cell proliferation, angiogenesis and fibroblast activity by shielding growth factors from proteolytic degradation [19,33]. Notably, topical sucralfate has shown an excellent safety profile in the available studies, with no significant adverse effects reported in either human clinical trials or animal models, further supporting its potential suitability for veterinary use [6,13,14,15,18,19,20,21,22,23,24,25,26,27,32,33,34,35,36,37,38,39,40,41,42,43,44,45,46,47,48,49].

Further comparative studies are needed to evaluate sucralfate against other topical agents currently used in veterinary medicine. These studies should include both macroscopic assessments—such as daily wound size measurements—and detailed immunohistochemical, histological and biomolecular analyses using skin biopsies. Investigating sucralfate’s mechanism of action in animals predisposed to exuberant granulation tissue production could yield valuable insights. By accelerating and strengthening the inflammatory phase, sucralfate may help to prevent the dysregulated proliferative phase, thereby shortening recovery times and reducing complications [51].

Wound healing in horses is further challenged by environmental factors such as fecal contamination, dirt, plant debris and foreign materials, which frequently lead to infections [49]. Common pathogens include *Pseudomonas*, *Enterococcus* and *Staphylococcus* species, all of which can delay wound closure [53]. Effective wound cleaning, appropriate dressing and the use of topical antimicrobials are essential to prevent biofilm formation. Preliminary studies suggest that sucralfate may exhibit both bacteriostatic and bactericidal properties against common skin pathogens [29]. Sucralfate’s activity across a wide pH range is particularly noteworthy [13]. While a pH below 6.5 is detrimental to wound healing, a mildly alkaline environment (pH < 7.5) does not impair healing [54]. Since the optimal pH for wound healing is around 7.5, understanding how sucralfate modulates pH dynamics could further support its use in creating favorable conditions for rapid tissue repair. Wound healing differs significantly between canine and feline species [5]. In cats, wound contraction is the primary closure mechanism, whereas, in dogs, epithelialization predominates [55]. Cats also display reduced skin vascularization, delayed immature collagen production and weaker healed tissue [5]. Additionally, granulation tissue forms peripherally before progressing centrally [5]. In contrast, dogs show more intense local perfusion, a faster inflammatory response and uniform granulation tissue production across the wound bed [5].

Sucralfate, through its growth factor binding capacity, can protect these molecules from enzymatic degradation, thereby enhancing local vascularization, fibroblast proliferation and granulation tissue formation [6,18,19]. Further research is warranted to determine whether sucralfate can accelerate wound healing in species with inherently slower healing, such as cats [55]. Comparative studies using macroscopic, histological, immunohistochemical and biomolecular techniques would be instrumental in evaluating its efficacy across different species and in comparison to existing treatments.

While numerous studies highlight the beneficial effects of topical sucralfate in cutaneous wound healing, there are several limitations of this study that might influence the overall judgement. The veterinary-specific literature remains based only on experimental animal models, with a significant lack of well-designed randomized clinical trials involving commonly encountered veterinary species.

Among the limited comparative studies available, Yuniati et al. (2021) [19] reported that sucralfate and platelet-rich plasma (PRP) showed comparable efficacy in promoting the healing of diabetic ulcers in rat models. Notably, the combination of sucralfate and PRP was found to be the most effective treatment strategy, significantly accelerating wound healing through enhanced fibroblast activity, angiogenesis and tissue remodeling. In addition, sucralfate has demonstrated superior outcomes compared to silver sulfadiazine in several experimental burn models, both in humans and animals [32,33,49], suggesting advantages in specific clinical conditions. However, despite these encouraging findings, comparative studies with other regenerative agents available in the market—such as medical honey [56,57], silver nanoparticles [58,59,60], stem cell-based therapies [61,62] and fluorescent light energy (FLE) [63,64]—are still lacking.

Without direct comparative trials, it remains difficult to determine whether sucralfate offers superior or equivalent benefits compared to these alternatives.

With the growing demand from pet owners for advanced clinical care, the development and validation of innovative, cost-effective treatments that expedite healing while minimizing complications is essential. This will not only improve clinical outcomes but also strengthen the trust between veterinarians and owners.

## 6. Conclusions

The extensive clinical evidence accumulated over the past years positions topical sucralfate as one of the most promising therapeutic agents for the management of mucocutaneous wounds in human medicine, thanks to its combined effects encompassing growth factor protection, anti-inflammatory activity and reepithelialization. Preclinical findings in animal models confirm that these mechanisms are biologically reproducible, offering a strong foundation for translational applications in veterinary medicine. However, the veterinary-specific literature remains poor and largely limited to experimental animals, with a notable lack of randomized clinical trials in animals commonly encountered in clinical practice. Despite this limitation, the available experimental data suggest that topical sucralfate may represent a novel and valuable therapeutic option, particularly for animals with delayed wound healing, abnormal inflammatory responses or a tendency to develop complications, such as exuberant granulation tissue.

Sucralfate’s ability to modulate the inflammatory and proliferative phases of the healing process, while protecting the skin microenvironment from enzymatic, chemical and microbial insults, makes it a valuable candidate for comparative wound management studies.

Given the species-specific complexities of wound healing in veterinary medicine, there is a need for controlled studies incorporating macroscopic, histological, immunohistochemical and biomolecular analyses. Such research could fill a critical scientific gap while opening up new options for the treatment of cutaneous wounds, combining therapeutic efficacy, safety and cost-effectiveness.

As veterinary medicine continues to evolve toward personalized and high-standard clinical care, topical sucralfate emerges as a versatile, affordable and evidence-supported agent to treat cutaneous wounds, improving healing outcomes, reducing complications and enhancing the mutual trust between animals and owners.

## Figures and Tables

**Table 1:** Summary of clinical studies on the topical use of sucralfate in human medicine.

Study (Author, Year, Type)	Condition	Protocol	Main Outcomes
Godhi et al., 2017, Randomized controlled trial [32]	Second-degree burn wounds	Topical sucralfate dressing vs. 1% silver sulfaziadine dressing	Faster granulation tissue formation compared to silver sulfadiazine. Comparable antimicrobial effectiveness.
Koshariya et al., 2018 Observational study [33]	Burn wounds	Topical sucralfate vs. topical silver sulfadiazine	Faster reepithelialization and granulation tissue formation, lower infection incidence and pain relief compared to silver sulfadiazine.
Saei et al., 2020, Randomized, double-blind, placebo-controlled clinical trial [34]	Radiotherapy-induced proctitis	Sucralfate ointment vs. placebo rectally	Reduced rectal bleeding, diarrhea and rectal pain compared to placebo.
Shum et al., 2024, Non-randomized clinical trial [35]	Radiotherapy-induced proctitis	Sucralfate enemas	Reduced rectal bleeding, decreased hospitalization, reduced need for invasive treatments.
Kouloulias et al., 2013, Non-randomized clinical trial [36]	Radiation dermatitis	Topical sucralfate humid gel (Skincol^®^, Erba, Italy)	Protective effect against dermatitis, reduced radiation-induced skin toxicity.
Rudiman et al., 2023, Systematic review and meta-analysis of randomized controlled trials [13]	Post-hemorrhoidectomy wounds	Topical sucralfate formulation	Reduced pain and analgesic consumption, improved wound healing.
Marik et al., 2024, Prospective observational study [37]	Hemorrhoidal symptoms	Sucralfate-based rectal ointment or suppositories	Reduction in hemorrhoidal symptoms, improved hemorrhoid grades.
Abdel Sattar et al., 2019, Randomized controlled trial [20]	Post-hemorrhoidectomy pain	Topical sucralfate formulation vs. placebo	Pain reduction and fast healing rate compared to placebo.
Vejdan et al., 2020, Randomized controlled trial [45]	Post-hemorrhoidectomy wounds	Topical sucralfate ointment vs. placebo	Significant pain reduction and analgesic consumption, accelerated healing.
Tumino et al., 2008, Randomized, double-blind, placebo-controlled trial [40]	Venous ulcers	Topical sucralfate cream vs. placebo	95.6% complete healing vs. 10.9% placebo, reduced pain and inflammation, faster ulcer reduction and granulation. Enhanced reepithelialization and angiogenesis.
Bhatmule et al., 2022, Comparative study [41]	Diabetic ulcers	Topical sucralfate formulation vs. saline dressing	Higher wound healing rate, reduction in ulcer size, reduced time to achieve complete healing.
Chatterjee et al., 2023, Open-label, randomized controlled trial [44]	Diabetic foot ulcers	Topical sucralfate + mupirocin ointment vs. mupirocin ointment	Healing time comparable to control group.
Ala et al., 2019, Prospective, double-blind, randomized, placebo-controlled trial [24]	Stage II pressure ulcers	Topical sucralfate gel vs. placebo	Healing time comparable to control group.
Pourandish et al., 2021, Case report [43]	Stage IV pressure ulcer	Topical sucralfate formulation + silver sulfadiazine	Ulcer improvement (stage II), reduction in ulcer size and tissue appearance.
Markham et al., 2000, Case series/clinical observation [39]	Infant irritant diaper dermatitis	Topical sucralfate ointment	Reduction in inflammation and fast healing rate.
Siupsinskiene et al., 2015, Randomized controlled trial [46]	Post-tonsillectomy or -adenotonsillectomy wounds	Topical sucralfate formulation vs. placebo	Reduced throat pain, odynophagia, otalgia. Reduced analgesic consumption and faster recovery.
Giua et al., 2021, Prospective, multicenter, observational study [48]	Hemorrhoidal disease	Topical rectal ointment with sucralfate and herbal extracts	Reduced pain, itching, bleeding and prolapse.

**Table 2:** Summary of experimental studies evaluating topical sucralfate in animal models of wound healing.

Study	Animal Model	Route/Formulation	Wound Type	Outcomes
Yildizhan et al., 2022 [18]	Rats	Topical application of 10% sucralfate cream	Full-thickness dorsal skin wounds, 2 cm diameter	Sucralfate significantly enhanced wound healing, with ↑ neovascularization, ↑ collagen organization, ↑ EGF expression and faster wound closure at days 7, 14 and 21 (*p* ≤ 0.05).
Le et al., 2023 [6]	Diabetic mice and pigs	Sucralfate-based microneedles delivering IL-4	Full-thickness excisional wounds (model-dependent)	Dual-action system promoted M2 macrophage polarization and growth factor stabilization, leading to fastest wound closure, ↑ angiogenesis and ↑ collagen deposition vs. controls.
Yuniati et al., 2023 [19]	Diabetic rats	Topical sucralfate, PRP or combination	Chronic diabetic ulcers (dimensions not specified)	Sucralfate alone ↑ fibroblast activity, ↑ angiogenesis, ↑ local growth factors, ↑ tissue remodeling, leading to improved contraction and reepithelialization.
Yaşar et al., 2023 [22]	Rats	Topical application of 10% sucralfate cream	Radiofrequency-induced burns	Early ↑ vasodilation and leukocyte chemotaxis; from day 14, ↑ angiogenesis, ↑ fibroblasts, ↑ collagen synthesis and ↑ reepithelialization. Thick granulation tissue by week 4.
Beheshti et al., 2013 [49]	Rats	Topical sucralfate cream vs. silver sulfadiazine	Second-degree thermal burns	Sucralfate group showed 100% wound closure vs. 91% with SSD and 76% with control. Histology revealed more complete epidermal regeneration and skin appendage formation.

## Data Availability

No new data were generated in the present study. The original contributions presented in this study are included in the article. Further inquiries can be directed to the corresponding author.

## References

[1] Harris-Tryon T.A., Grice E. (2022). Microbiota and maintenance of skin barrier function. Science.

[2] Borena B.M., Martens A., Broeckx S.Y., Meyer E., Chiers K., Duchateau L., Spaas J.H. (2015). Regenerative skin wound healing in mammals: State-of-the-art on growth factor and stem cell-based treatments. Cell. Physiol. Biochem..

[3] Sorg H., Tilkorn D.J., Hager S., Hauser J., Mirastschijski U. (2017). Skin wound healing: An update on the current knowledge and concepts. Eur. Surg. Res..

[4] Tambella A.M., Attili A.R., Dupre G., Cantalamessa A., Martin S., Cuteri V., Marcazzan S., Del Fabbro M. (2018). Platelet-rich plasma to treat experimentally-induced skin wounds in animals: A systematic review and meta-analysis. PLoS ONE.

[5] Lux C.N. (2022). Wound healing in animals: A review of physiology and clinical evaluation. Vet. Dermatol..

[6] Le Z., Ramos M.C., Shou Y., Li R.R., Cheng H.S., Jang C.J., Liu L., Xue C., Li X., Liu H. (2024). Bioactive sucralfate-based microneedles promote wound healing through reprogramming macrophages and protecting endogenous growth factors. Biomaterials.

[7] Harman R.M., Theoret C.L., Van de Walle G.R. (2021). The horse as a model for the study of cutaneous wound healing. Adv. Wound Care.

[8] Hill P.B., Lo A., Eden C.A.N., Huntley S., Morey V., Ramsey S., Richardson C., Smith D.J., Sutton C., Taylor M.D. (2006). Survey of the prevalence, diagnosis and treatment of dermatological conditions in small animals in general practice. Vet. Rec..

[9] Hill P. (2002). Small Animal Dermatology: A Practical Guide to the Diagnosis and Management of Skin Diseases in Dogs and Cats.

[10] Marsella R., Olivry T., Carlotti D.N. (2011). Current evidence of skin barrier dysfunction in human and canine atopic dermatitis. Vet. Dermatol..

[11] Woldemeskel M. (2019). Nutraceuticals in Dermatological Disorders. Nutraceuticals in Veterinary Medicine.

[12] Aisa J., Parlier M. (2022). Local wound management: A review of modern techniques and products. Vet. Dermatol..

[13] Rudiman R., Hanafi R.V., Evan C., Halim F. (2023). The efficacy of topical sucralfate in improving pain and wound healing after haemorrhoidectomy procedure: A systematic review, meta-analysis, and meta-regression of randomised clinical trials. Int. Wound J..

[14] Abtahi-Naeini B., Saffaei A., Sabzghabaee A.M., Amiri R., Hosseini N.S., Niknami E., Dehghani S. (2022). Topical sucralfate for treatment of mucocutaneous conditions: A systematic review on clinical evidences. Dermatol. Ther..

[15] Masuelli L., Tumino G., Turriziani M., Modesti A., Bei R. (2010). Topical use of sucralfate in epithelial wound healing: Clinical evidence and molecular mechanisms of action. Recent Pat. Inflamm. Allergy Drug Discov..

[16] Tolbert K., Stubbs E. (2024). Rational use of gastroprotectants in cats: An evidence-based approach. J. Feline Med. Surg..

[17] Vokes J., Lovett A., Sykes B. (2023). Equine gastric ulcer syndrome: An update on current knowledge. Animals.

[18] Yildizhan E., Ulger B.V., Akkus M., Akinci D., Basol O. (2022). Comparison of topical sucralfate with dexpanthenol in rat wound model. Int. J. Exp. Pathol..

[19] Yuniati R., Innelya I., Rachmawati A., Charlex H.J.M., Rahmatika A., Khrisna M.B., Mundhofir F.E.P., Seno T.N., Kristina T.N. (2021). Application of topical sucralfate and topical platelet-rich plasma improves wound healing in diabetic ulcer rats wound model. J. Exp. Pharmacol..

[20] Abdel Sattar A.M., Al Batanony A.A., Al Khateeb Y.M. (2019). Evaluation of the role of sucralfate cream in decreasing pain intensity and improving healing following open hemorrhoidectomy: A randomized controlled study. Menoufia Med. J..

[21] Gutta S.H., Kotennavar M.S., Patil A., Benakatti R., Jaju P., Savanth M. (2023). Topical Solutions for Chronic Lower Limb Ulcers: A Comparative Study of Sucralfate and 5% Povidone-Iodine. Int. J. Sci. Study.

[22] Yaşar Ş., Yaşar B., Yörüsün A., Güneş P., Kayadibi H., Ercin Z., Aytekin S. (2020). A Controlled Study to Examine the Effect of Topical Sucralfate on Radiofrequency-induced Burn Wounds in Rats. Wound Manag. Prev..

[23] Bryan N., Ahswin H., Smart N., Bayon Y., Wohlert S., Hunt J.A. (2012). Reactive oxygen species (ROS)—A family of fate deciding molecules pivotal in constructive inflammation and wound healing. Eur. Cells Mater..

[24] Ala S., Saeedi M., Gholipour A., Ahmadi M., Asoodeh A., Shiva A. (2019). Effectiveness of topical sucralfate in the management of pressure ulcer in hospitalized patients: A prospective, randomized, placebo-controlled trial. Am. J. Ther..

[25] Freeman S.B., Markwell J.K. (1992). Sucralfate in alleviating post-tonsillectomy pain. Laryngoscope.

[26] Özcan M., Altuntasl A., Ünal A., Nalcla Y., Aslan A. (1998). Sucralfate for posttonsillectomy analgesia. Otolaryngol.–Head Neck Surg..

[27] Gupta P.J., Heda P.S., Kalaskar S., Tamaskar V.P. (2008). Topical sucralfate decreases pain after hemorrhoidectomy and improves healing: A randomized, blinded, controlled study. Dis. Colon Rectum.

[28] Tryba M., Mantey-Stiers F. (1987). Antibacterial activity of sucralfate in human gastric juice. Am. J. Med..

[29] Yen T., Boord M.J., Ghubash R., Blondeau J.M. (2018). A pilot study investigating the in vitro efficacy of sucralfate against common veterinary cutaneous pathogens. J. Small Anim. Pract..

[30] Li J., Xie J., Wang Y., Li X., Yang L., Zhao M., Chen C. (2024). Development of Biomaterials to Modulate the Function of Macrophages in Wound Healing. Bioengineering.

[31] Linata A.M., Fawzy A. (2024). Does The Scientific Evidence Support the Idea of Promoting Sucralfate as The First Aid Topical Management for Burn Wound? A Literature Review. Int. J. Med. Sci. Clin. Res. Stud..

[32] Godhi A.S., Ram P., Powar R. (2017). Efficacy of topical sucralfate versus silver sulfadiazine in the management of burns: A 1-year randomized controlled trial. J. West Afr. Coll. Surg..

[33] Koshariya M., Shitole A., Agarwal V., Dave S. (2018). Role of topical Sucralfate in healing of burn wounds. Int. Surg. J..

[34] Saei S., Sahebnasagh A., Ghasemi A., Akbari J., Alipour A., Lashkardoost H., Joybari A.Y., Dadgar N.F., Ala S., Salehifar E. (2020). Efficacy of sucralfate ointment in the prevention of acute proctitis in cancer patients: A randomized controlled clinical trial. Casp. J. Intern. Med..

[35] Shum N.F., Choi H.K., Wei R., Dominic C.C.F. (2024). Evaluating self-administered sucralfate enemas for reducing rectal bleeding in patients with radiation proctitis. Cancer Nurs. Pract..

[36] Kouloulias V., Asimakopoulos C., Tolia M., Filippou G., Platoni K., Dilvoi M., Beli I., Georgakopoulos J., Patatoukas G., Kelekis N. (2013). Sucralfate gel as a radioprotector against radiation induced dermatitis in a hypo-fractionated schedule: A non-randomized study. Hippokratia.

[37] Marik A.R., Miklós I., Csukly G., Hársfalvi P., Novák A. (2024). Effectiveness and tolerability of rectal ointment and suppositories containing sucralfate for hemorrhoidal symptoms: A prospective, observational study. Int. J. Color. Dis..

[38] Lamb S., Coulter L., Hudson J. (2016). Is topical sucralfate an effective therapy for noncandidal diaper rash?. Evid.-Based Pract..

[39] Markham T., Kennedy F., Collins P. (2000). Topical sucralfate for erosive irritant diaper dermatitis. Arch. Dermatol..

[40] Tumino G., Masuelli L., Bei R., Simonelli L., Santoro A., Francipane S. (2008). Topical treatment of chronic venous ulcers with sucralfate: A placebo controlled randomized study. Int. J. Mol. Med..

[41] Bhatmule A., Janugade H.B., Nangare N. (2022). A competitive study of topical sucralfate and ordinary saline for diabetic ulcer dressing. J. Pharm. Negat. Results.

[42] Chatterjee S., Sen S., Hazra A., Das A.K. (2019). Randomized controlled trial of topical mupirocin versus mupirocin with sucralfate combination in chronic skin ulcers. Indian J. Pharmacol..

[43] Pourandish Y., Mehrabi F., Veldani N.A., Tabar R.M. (2021). Pressure ulcer healing by daily topical sucralfate and silver sulfadiazine: A case report study. J. Nurs. Midwifery Sci..

[44] Chatterjee N., Ekka N.M., Mahajan M., Kumar B., Kumar N., Zia A., Devarajan A., Kujur A.D., Sinha D.K. (2023). Effectiveness of Topical Sucralfate in the Management of Diabetic Foot Ulcers: An Open-Labeled Randomized Study. Cureus.

[45] Vejdan A.K., Khosravi M., Amirian Z., Daneshmand M., Babak B., Samira K., Azin S., Kosar S., Razie K. (2020). Evaluation of the efficacy of topical sucralfate on healing haemorrhoidectomy incision wounds and reducing pain severity: A randomised clinical trial. Int. Wound J..

[46] Siupsinskiene N., Žekonienė J., Padervinskis E., Žekonis G., Vaitkus S. (2015). Efficacy of sucralfate for the treatment of post-tonsillectomy symptoms. Eur. Arch. Oto-Rhino-Laryngol..

[47] Suparakchinda C., Rawangban W. (2023). Effectiveness of Sucralfate comparing to normal saline as an oral rinse in pain reduction and wound healing promotion in oral surgery. Laryngoscope Investig. Otolaryngol..

[48] Giua C., Minerba L., Piras A., Floris N., Romano F. (2021). The effect of sucralfate-containing ointment on quality of life in people with symptoms associated with haemorrhoidal disease and its complications: The results of the EMOCARE survey. Acta Biomed. Atenei Parm..

[49] Beheshti A., Shafigh Y., Zangivand A.A., Samiee-Rad F., Hassanzadeh G., Shafigh N. (2013). Comparison of topical sucralfate and silver sulfadiazine cream in second degree burns in rats. Adv. Clin. Exp. Med..

[50] Ribeiro G., Carvalho L., Borges J., Prazeres J. (2024). The Best Protocol to Treat Equine Skin Wounds by Second Intention Healing: A Scoping Review of the Literature. Animals.

[51] Eggleston R.B. (2018). Wound management: Wounds with special challenges. Vet. Clin. Equine Pract..

[52] Hamed M.A., Abouelnasr K.S., El-Adl M., Elfadl E.A.A., Farag A., Lashen S. (2019). Effectiveness of allogeneic platelet-rich fibrin on second-intention wound healing of experimental skin defect in distal limb in donkeys (Equus asinus). J. Equine Vet. Sci..

[53] Marchant K., Hendrickson D.A., Pezzanite L.M. (2024). Review of the role of biofilms in equine wounds: Clinical indications and treatment strategies. Equine Vet. Educ..

[54] Kruse C.R., Singh M., Targosinski S., Sinha I., Sørensen J.A., Eriksson E., Nuutila K. (2017). The effect of pH on cell viability, cell migration, cell proliferation, wound closure, and wound reepithelialization: In vitro and in vivo study. Wound Repair Regen..

[55] Bohling M.W., Henderson R.A., Swaim S.F., Kincaid S.A., Wright J.C. (2004). Cutaneous wound healing in the cat: A macroscopic description and comparison with cutaneous wound healing in the dog. Vet. Surg..

[56] Tashkandi H. (2021). Honey in wound healing: An updated review. Open Life Sci..

[57] Zainuddin A.N.Z., Mustakim N.N., Rosemanzailani F.A., Fadilah N.I.M., Maarof M., Fauzi M.B. (2025). A Comprehensive Review of Honey-Containing Hydrogel for Wound Healing Applications. Gels.

[58] Rybka M., Mazurek L., Konop M. (2023). Beneficial effect of wound dressings containing silver and silver nanoparticles in wound healing—From experimental studies to clinical practice. Life.

[59] Jangid H., Singh S., Kashyap P., Singh A., Kumar G. (2024). Advancing biomedical applications: An in-depth analysis of silver nanoparticles in antimicrobial, anticancer, and wound healing roles. Front. Pharmacol..

[60] Popescu I., Constantin M., Solcan G., Ichim D.L., Rata D.M., Horodincu L., Solcan C. (2023). Composite Hydrogels with Embedded Silver Nanoparticles and Ibuprofen as Wound Dressing. Gels.

[61] Farabi B., Roster K., Hirani R., Tepper K., Atak M.F., Safai B. (2024). The Efficacy of Stem Cells in Wound Healing: A Systematic Review. Int. J. Mol. Sci..

[62] Gao M., Guo H., Dong X., Wang Z., Yang Z., Shang Q., Wang Q. (2024). Regulation of inflammation during wound healing: The function of mesenchymal stem cells and strategies for therapeutic enhancement. Front. Pharmacol..

[63] Scapagnini G., Marchegiani A., Rossi G., Zago M., Jowarska J., Wael M., Campbell S.E., Schiffman Z., Buonamici E., Garvao R. Management of all three phases of wound healing through the induction of fluorescence biomodulation using fluorescence light energy. Proceedings of the Photonic Diagnosis Treatment Infections Inflammatory Diseases II.

[64] Nikolis A., Grimard D., Pesant Y., Scapagnini G., Vezina D.A. (2016). A prospective case series evaluating the safety and efficacy of the Klox BioPhotonic System in venous leg ulcers. Chronic Wound Care Manag. Res..

